# Presacral lymph node recurrence of rectal intramucosal adenocarcinoma after endoscopic mucosal resection: a case report

**DOI:** 10.1186/s40792-020-00836-7

**Published:** 2020-04-21

**Authors:** Taichi Horino, Yukiharu Hiyoshi, Yuji Miyamoto, Naoya Yoshida, Hideo Baba

**Affiliations:** Department of Gastroenterological Surgery, Graduate School of Medical Sciences, Kumamoto University, 1-1-1 Honjo, Kumamoto, 860-8556 Japan

**Keywords:** Colorectal cancer, Intramucosal adenocarcinoma, Endoscopic mucosal resection, Lymph node recurrence

## Abstract

**Background:**

The recurrence of endoscopically resected intramucosal colorectal cancer (CRC) is quite rare, and data regarding metastasis in intramucosal tumors are still lacking. We herein report a case of presacral lymph node recurrence of intramucosal rectal cancer after curative endoscopic resection.

**Case presentation:**

A 53-year-old man underwent endoscopic mucosal resection (EMR) for rectal intramucosal adenocarcinoma. Thirty-nine months after the procedure, follow-up computed tomography (CT) revealed a swollen anterior sacral lymph node with an abnormal fluorodeoxyglucose (FDG) uptake on positron emission tomography (PET). He underwent laparoscopic low anterior resection (LAR) and was discharged on postoperative day 11 without any complications. The pathological examination confirmed solitary lymph node metastasis (moderately differentiated adenocarcinoma) without a residual tumor in the rectal epithelium. We diagnosed him with lymph node metastasis of rectal cancer. Pathological examination of the resected lymph node confirmed moderately differentiated adenocarcinoma. He has not experienced any re-recurrence in the 6 months since surgery.

**Conclusions:**

This is a rare case of local lymph node recurrence of intramucosal rectal cancer after successful EMR that was salvaged with surgery. Surveillance after successful endoscopic resection of rectal cancer using both endoscopy and CT is necessary.

## Introduction

Colorectal cancer (CRC) with invasion limited to the lamina propria (LP) is defined as intramucosal carcinoma. According to the current consensus, intramucosal CRC is not expected to metastasize because colonic LP lacks lymphatics [1], and endoscopic resection is regarded as adequate treatment. However, recent reports have described local or distant metastasis after curative resection for intramucosal rectal cancer [2–5]. Data regarding metastasis in intramucosal tumors are still lacking, and the metastatic potential of intramucosal CRC remains unclear.

We herein report a case of presacral lymph node recurrence of intramucosal rectal cancer 39 months after curative endoscopic resection.

## Case presentation

A 53-year-old man who had a history of type 2 diabetes mellitus and dyslipidemia submitted to regular surveillance. He was diagnosed with ascending colon cancer 9 months earlier, and he underwent laparoscopic right hemicolectomy with D3 lymphadenectomy. The pathological diagnosis was moderately differentiated tubular adenocarcinoma of the ascending colon, infiltrating over the serosa with intermediate lymph node metastasis (pT4N2M0, UICC 7th Ed.). The resected specimen was margin-negative. We performed adjuvant chemotherapy with CapOX (capecitabine 2000 mg/m^2^/day, oxaliplatin 130 mg/m^2^) for 6 months. His postoperative course was uneventful, and the patient did not experience any adverse events.

**Fig. 1:**
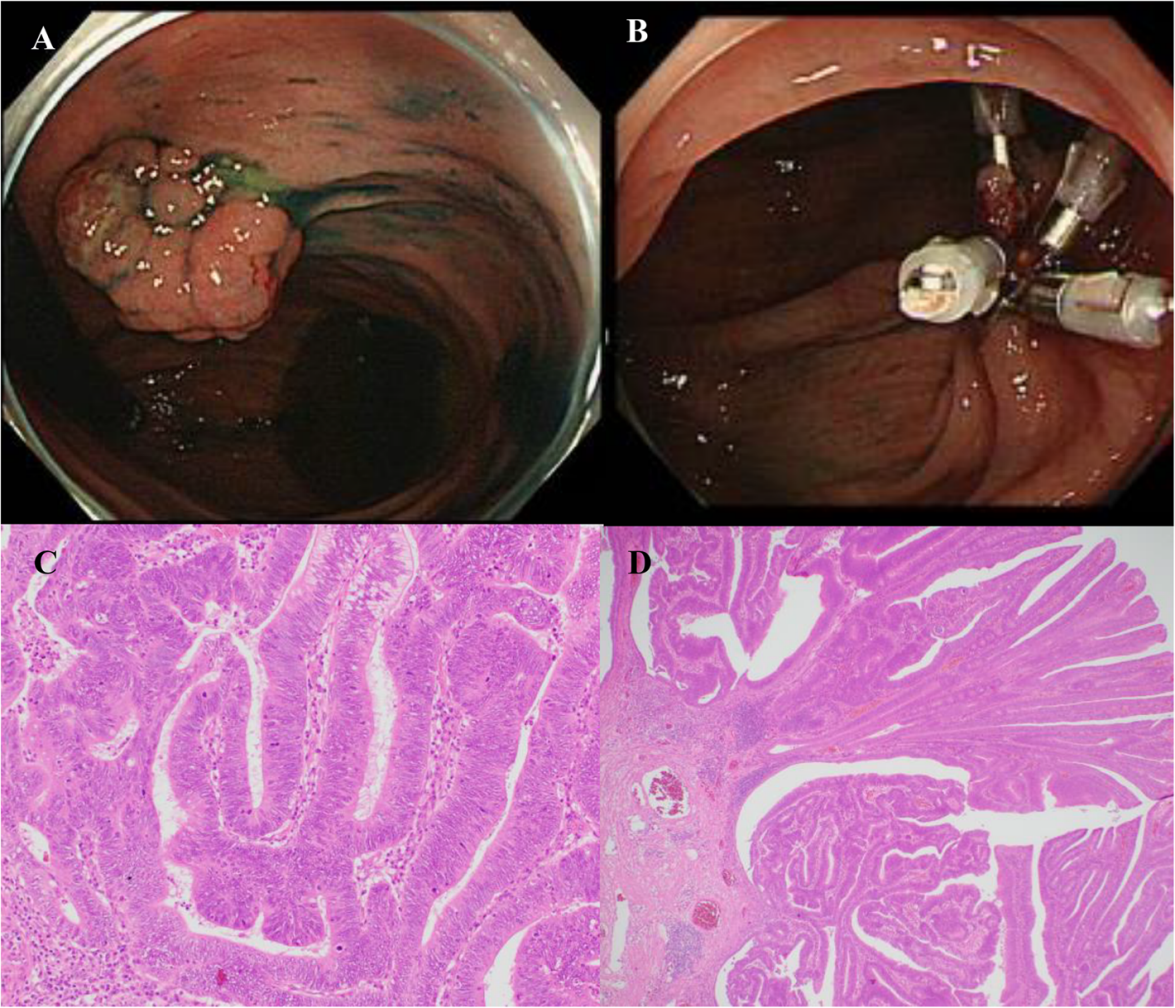
Endoscopic mucosal resection for a rectal polyp. **a** A 23-mm sessile polyp was located at the upper rectum. **b** Clips were placed endoscopically after endoscopic mucosal resection. **c** A pathological examination revealed moderately differentiated tubular carcinoma in adenoma (hematoxylin and eosin staining). **d** Both the horizontal and vertical margins were negative for malignancy (hematoxylin and eosin staining)

During regular surveillance, endoscopy showed a 23-mm sessile polyp at the superior rectum 9 months after the surgery. Endoscopic mucosal resection (EMR) was successfully performed, and the pathological examination revealed moderately differentiated tubular carcinoma in adenoma. There were no findings of submucosal invasion or tumor budding. Both the horizontal and vertical margins were negative for malignancy (Fig. 1). Lymphovascular invasion was not detected by immunohistochemical examinations using D2-40 and Victoria blue staining.

**Fig. 2:**
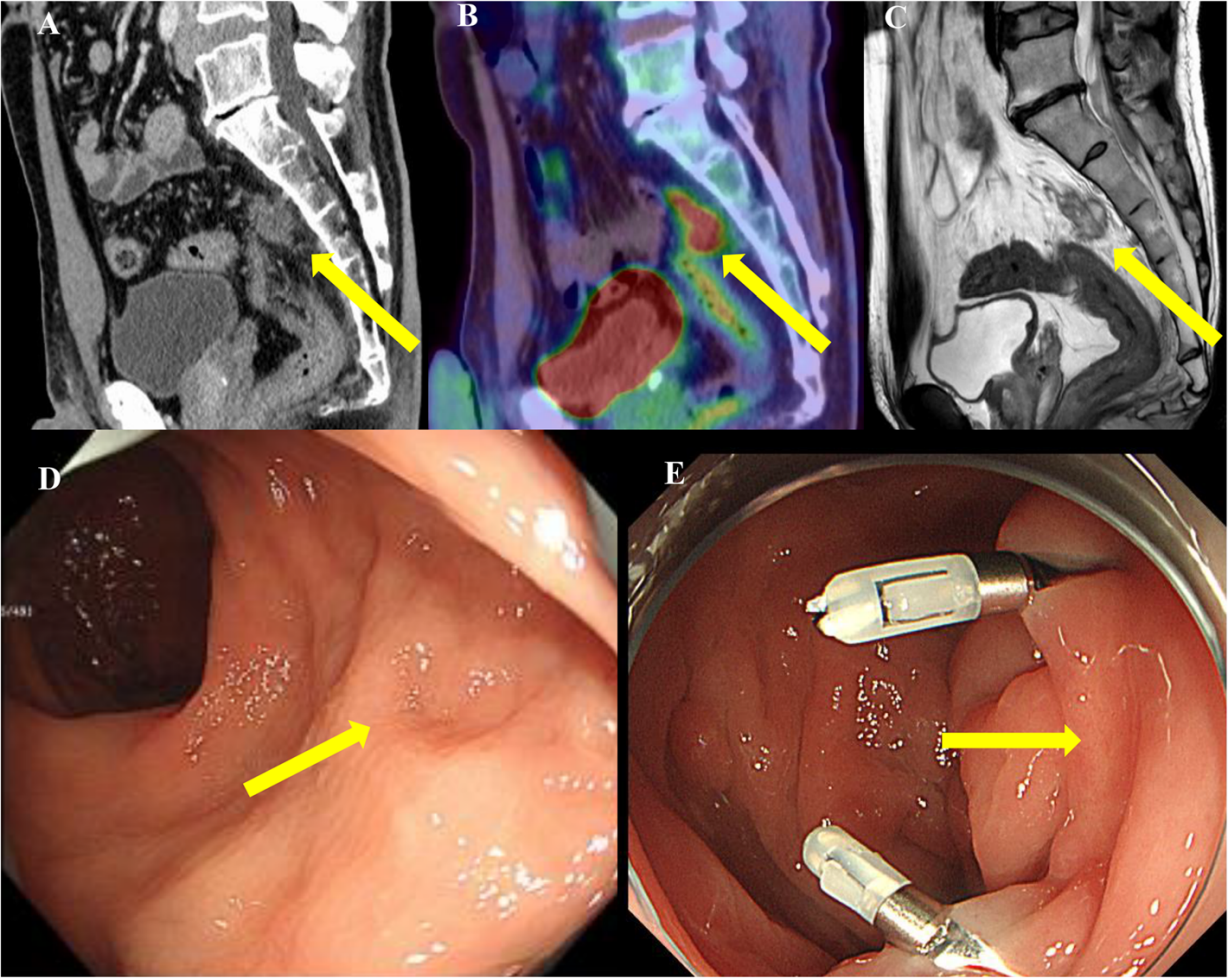
Preoperative imaging showing the recurrent tumor at the presacral area. **a**, **b** PET-CT shows a tumor with an abnormal FDG uptake at the presacral area. **c** MRI shows no evidence of invasion to the rectum or sacrum. **d** Endoscopy showed the EMR scar at the upper rectum, but no evidence of new mucosal lesions. **e** Clipping and marking near the scar were performed endoscopically

Thirty-nine months after EMR, CT, as postoperative surveillance for ascending colon cancer, showed a mass lesion at the presacral area. Fluorodeoxyglucose (FDG)-positron emission tomography (PET) showed an abnormal FDG uptake at the lesion. We obtained no evidence of rectal infiltration on magnetic resonance imaging (MRI). No other findings of metastasis or recurrence were found by imaging. Endoscopy revealed the EMR scar at the upper rectum, but we noted no new mucosal lesions (Fig. 2). Since the recurrent tumor was in the mesorectum near the EMR scar and was solitary, we diagnosed him with presacral lymph node recurrence of rectal cancer.

**Fig. 3:**
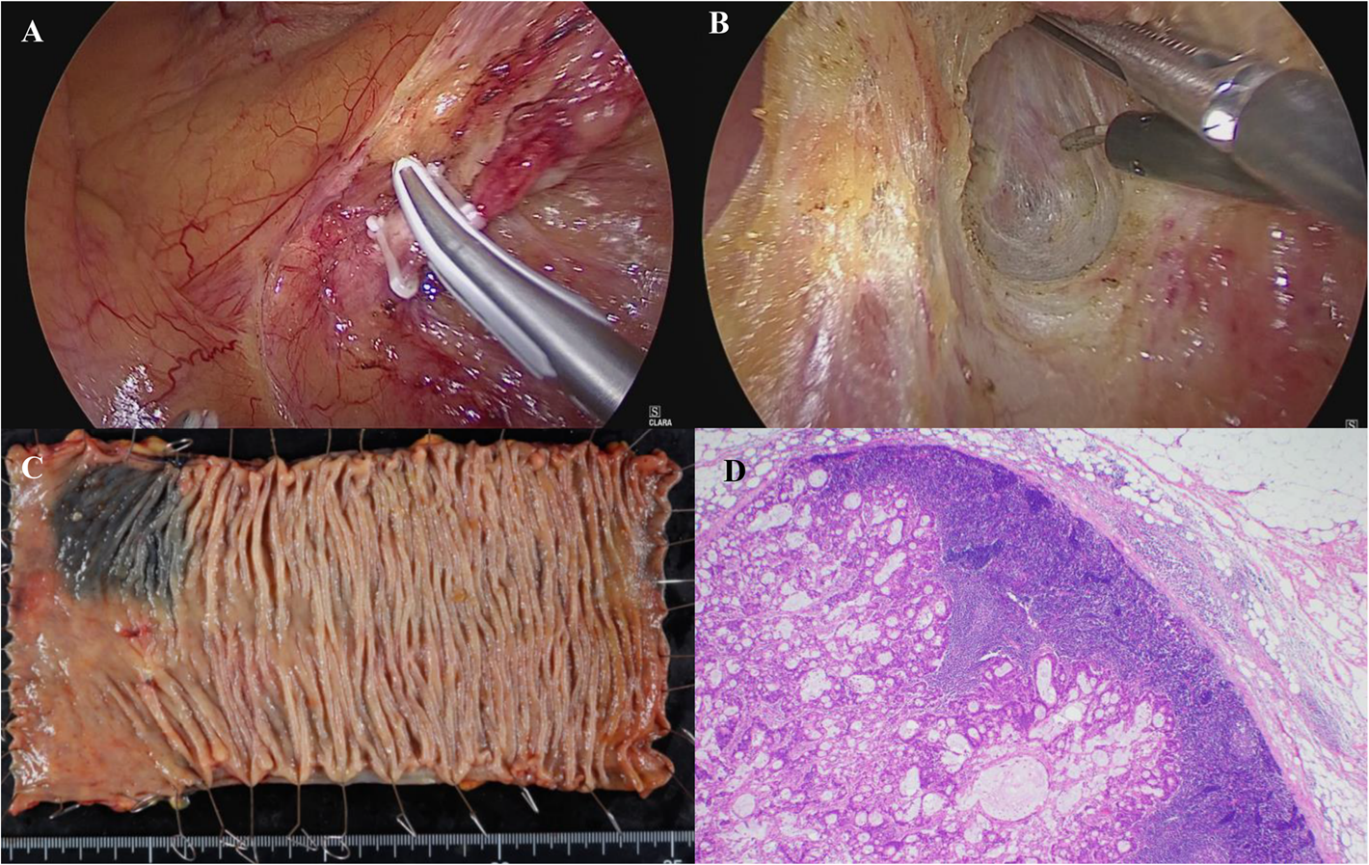
Laparoscopic low anterior resection for the recurrent tumor. **a** The root of the inferior mesenteric artery was clipped and cut (D3 lymph node dissection). **b** The recurrent tumor was located at the mesorectum in the presacral area. There was no intraoperative finding of invasion or adhesion to the sacrum. **c** Macroscopically, there were no abnormal findings on mucosa. **d** A pathological examination confirmed solitary lymph node metastasis (moderately differentiated adenocarcinoma) of rectal cancer

The patient underwent LAR with D3 lymphadenectomy (Fig. 3). The recurrent tumor was located at the mesorectum in the presacral area. Fortunately, there was no intraoperative finding of invasion or adhesion to the sacrum. The postoperative course was uneventful, and he was discharged from our hospital on postoperative day 11. Macroscopically, there were no abnormal findings in the mucosa of the resected specimen. The pathologic examination confirmed solitary lymph node metastasis (moderately differentiated adenocarcinoma) with vascular involvement. There was no residual tumor in the rectal epithelium and no metastasis in other resected lymph nodes. The recurrent site was completely removed by surgery. We simply followed him up without adjuvant chemotherapy, and he has not experienced any re-recurrence in the 6 months since surgery.

## Discussion

Recurrence of intramucosal colorectal adenocarcinoma is extremely rare. Because colonic LP is commonly thought to lack lymphatics [1], intramucosal CRC was believed not to metastasize. However, such recurrence has already been reported in five cases in the English-language literature [2–5]. Lee et al. reported two cases of recurrence after endoscopic submucosal dissection (ESD) [2]. The recurrent sites were the same as the ESD sites in both cases. Shia et al. reported the local recurrence of rectal adenocarcinoma after LAR [3]. In those cases, an endoscopic examination was useful for diagnosing recurrence.

However, Seo et al. reported a case of recurrence at the perirectal lymph nodes after ESD that was revealed by CT [4]. Lee et al. reported a case of recurrence at the common hepatic lymph node after LAR [5]. In those cases, there were no abnormal findings on an endoscopic examination, just as in our case. According to previous reports, CT played an important role in confirming the diagnosis of local recurrence in cases without endoscopic abnormalities [4, 5]. Therefore, we recommend clinical follow-up with systemic imaging, including CT, be performed after successful endoscopic resection of colorectal adenocarcinoma.

According to the Japanese Society for Cancer of the Colon and Rectum (JSCCR) Guidelines for the treatment of CRC, clinical surveillance after endoscopic resection of early colorectal cancer is recommended [6]. The recommended surveillance includes endoscopic examinations, imaging studies (such as CT), and tumor marker evaluations. Ikematsu et al. reported that the recurrence rate of endoscopically resected submucosal rectal adenocarcinoma without the need for additional surgical treatment was 6.3% [7]. Oka et al. suggested strict surveillance for 3 years after endoscopic resection for submucosal CRC [8]. Previous reports of recurrence of intramucosal colorectal adenocarcinoma showed that the period until recurrence ranges from 17 to 34 months [2–5]. However, we detected presacral lymph node recurrence of rectal cancer 39 months after EMR. This suggests that such patients need to be followed for more than 3 years after endoscopic resection with endoscopic examinations and systemic imaging (e.g., once yearly for 5 years).

Recently, the use of ctDNA (circulating tumor deoxyribonucleic acid) in the diagnosis of recurrence of CRC has been suggested. Monitoring ctDNA is a minimally invasive procedure and can be performed repeatedly at short intervals. Benešová et al. reported that cases of R0 surgery that displayed ctDNA postoperatively were diagnosed with recurrent CRC after 6 months [9]. Due to its potential, this method is attracting many scientists, and it has started to be applied in several clinical trials [10]. In the future, the monitoring of ctDNA might become a viable tool for predicting recurrence after curative resection of CRC.

The mechanism underlying the recurrence of intramucosal colorectal adenocarcinoma after resection remains unclear. However, there are some theories regarding the etiology of such recurrence. Tajika et al. suggested that implantation of malignant cells from the endoscopic resection site leads to recurrence [11]. However, while this might induce recurrence in the gastrointestinal tract, local lymph node recurrence as in our case would probably not occur. Fenoglio et al. noted that lymphatics have been shown to be present in the deep mucosa and within the muscularis mucosa [12]. It is therefore feasible that intramucosal adenocarcinoma might gain access to the intramucosal lymphatics and thereby metastasize. Shia et al. suggested that focal lymphatic tumor invasion at the base of the mucosa might be a possible route for metastasis in intramucosal colorectal carcinoma [3]. In our case, although there was no evidence of submucosal invasion of the tumor, we cannot exclude the possibility that we missed some pathologic findings, including focal submucosal invasive cancer, which might have led to recurrence. We need more clinical experience to verify the etiology.

According to the JSCCR Guidelines for the treatment of CRC, surgical resection is recommended for local recurrence of rectal cancer when curative resection is considered possible [6]. We should consider the surgical stress, risk, and postoperative quality of life before deciding on the indication of resection. In our case, the performance status of the patient was quite good at the time of recurrence. We confirmed that the local lymph node recurrence had been resected successfully by the operation. In addition, he was already treated with CapOX therapy as adjuvant chemotherapy for ascending colon cancer; hence, we simply followed the patient up without adjuvant chemotherapy. We will continue to perform careful surveillance of this patient.

## Conclusions

We encountered a rare case of local recurrence of lymph node in a patient who previously underwent successful EMR for intramucosal rectal adenocarcinoma. The recurrent site was successfully removed by surgery. This case highlights the possibility of recurrence during surveillance after endoscopic resection of intramucosal colorectal adenocarcinoma. We recommend performing prudent postoperative clinical surveillance including systemic imaging, especially CT.

## Data Availability

All data generated or analyzed during this study are included in this published article.

## Abbreviations

CRC: Colorectal cancer
EMR: Endoscopic mucosal resection
CT: Computed tomography
FDG: Fluorodeoxyglucose
PET: Positron emission tomography
LAR: Laparoscopic anterior resection
LP: Lamina propria
UICC: Union for International Cancer Control
CapOX: Capecitabine and oxaliplatin
MRI: Magnetic resonance imaging
ESD: Endoscopic submucosal dissection
JSCCR: Japanese Society for Cancer of the Colon and Rectum
ctDNA: Circulating tumor deoxyribonucleic acid
